# Role of ADAM10 and ADAM17 in the Regulation of Keratinocyte Adhesion in Pemphigus Vulgaris

**DOI:** 10.3389/fimmu.2022.884248

**Published:** 2022-06-30

**Authors:** Daniela Kugelmann, Maresa Anders, Anna M. Sigmund, Desalegn T. Egu, Ramona A. Eichkorn, Amir S. Yazdi, Miklós Sárdy, Michael Hertl, Dario Didona, Takashi Hashimoto, Jens Waschke

**Affiliations:** ^1^ Chair of Vegetative Anatomy, Faculty of Medicine, Ludwig-Maximilians-Universität (LMU) Munich, Munich, Germany; ^2^ Department of Dermatology, University Medical Center Tübingen, Eberhard Karls-University, Tübingen, Germany; ^3^ Department of Dermatology, Rheinisch-Westfälische Technische Hochschule (RWTH) Aachen, Aachen, Germany; ^4^ Department of Dermatology, Venereology and Dermatooncology, Semmelweis University, Budapest, Hungary; ^5^ Department of Dermatology and Allergy, University Hospital, Ludwig-Maximilians-Universität (LMU) Munich, Munich, Germany; ^6^ Department of Dermatology and Allergology, Philipps University of Marburg, Marburg, Germany; ^7^ Department of Dermatology, Graduate School of Medicine, Osaka City University, Osaka, Japan

**Keywords:** a disintegrin and metalloproteinase 10 (ADAM10), a disintegrin and metalloproteinase 17 (ADAM17), cell cohesion, desmoglein 3 (Dsg3), hyperadhesion, pemphigus vulgaris (PV)

## Abstract

The severe autoimmune blistering disease Pemphigus vulgaris (PV) is mainly caused by autoantibodies (IgG) against desmoglein (Dsg) 3 and Dsg1. The mechanisms leading to the development of blisters are not fully understood, but intracellular signaling seems to play an important role. Sheddases ADAM10 and ADAM17 are involved in the turnover of the desmosomal cadherin Dsg2 and ADAM10 has been shown to contribute to acantholysis in a murine pemphigus model. In the present study, we further examined the role of ADAM10 and ADAM17 both in keratinocyte adhesion and in the pathogenesis of PV. First, we found that inhibition of ADAM10 enhanced adhesion of primary human keratinocytes but not of immortalized keratinocytes. In dissociation assays, inhibition of ADAM10 shifted keratinocyte adhesion towards a hyperadhesive state. However, ADAM inhibition did neither modulate protein levels of Dsg1 and Dsg3 nor activation of EGFR at Y1068 and Y845. In primary human keratinocytes, inhibition of ADAM10, but not ADAM17, reduced loss of cell adhesion and fragmentation of Dsg1 and Dsg3 immunostaining in response to a PV1-IgG from a mucocutaneous PV patient. Similarly, inhibition of ADAM10 in dissociation assay decreased fragmentation of primary keratinocytes induced by a monoclonal antibody against Dsg3 and by PV-IgG from two other patients both suffering from mucosal PV. However, such protective effect was not observed in both cultured cells and *ex vivo* disease models, when another mucocutaneous PV4-IgG containing more Dsg1 autoantibodies was used. Taken together, ADAM10 modulates both hyperadhesion and PV-IgG-induced loss of cell adhesion dependent on the autoantibody profile.

## Introduction

Pemphigus vulgaris (PV) is an autoimmune dermatosis characterized by intraepithelial blistering in the skin and mucous membranes (1, 2). The pathogenic autoantibodies causing loss of keratinocyte cohesion are mainly directed against the desmosomal cadherins desmoglein 3 (Dsg3) and desmoglein 1 (Dsg1) (3, 4). In general, anti-Dsg3 autoantibodies lead to a mucosal phenotype characterized by blisters in mucous membranes only whereas in mucocutaneous PV additional anti-Dsg1 antibodies induce blistering in the skin also (5).

Beside direct inhibition of the interaction of demosomal cadherins, the antibody binding has been shown to trigger intercellular signaling pathways, which indirectly result in loss of desmoglein-mediated interaction and thus intraepidermal blistering. Kinases such as p38MAPK, PLC, ERK, and Src are activated by autoantibody binding and contribute to alterations of the keratin cytoskeleton and desmoglein internalization (6–15). Nevertheless, the exact mechanisms causing blisters are not fully understood, yet.

Membrane-anchored sheddases such as the a disintegrin and metalloprotease (ADAM) 10 and ADAM17, reside at cell-cell contacts and are involved in cell-adhesion, in addition to their proteolytic activity (16, 17), while their role is not entirely clear. ADAM10 and ADAM17 are well characterized metalloproteases and have a wide variety of functions, including cell-migration, fertilization, neural homeostasis and neurodegenerative diseases such as Alzheimer’s disease. They also play a role in the dysfunction of immune system and cancer (18–22), and in many developmental processes such as neurogenesis, angiogenesis and heart development (17). Deletion of ADAM10 and ADAM17 in mice is lethal in embryos (17). Both ADAMs cleave a broad spectrum of substrates such as cytokines and their receptors, growth factors, and cell adhesion molecules (17, 23, 24). Further, ADAM10 was shown to cleave various cadherins, such as N-cadherin, VE-cadherin and E-cadherin, as well as desmoglein 2 (Dsg2), which is also a substrate of ADAM17 (17, 24). In this context, there is evidence that both ADAM10 and E-cadherin are involved in the pathogenesis of eczematous inflammatory disorders and autoimmune dermatosis psoriasis (25–28). ADAMs are also involved in shedding of BP180 (180-kd bullous pemphigoid antigen) and therefore contribute to supradermal blister formation in bullous pemphigoid (29). ADAM10 has also been linked to acantholysis in a murine pemphigus mouse model (30), although the mechanism is not fully understood. In this study, we demonstrate for the first time a protective role of inhibition of ADAM10 in PV-IgG induced loss of intercellular adhesion in cultured human keratinocytes *in vitro*. However, the effect of ADAM10 appeared to depend on the antibody profile, because ADAM10 inhibition was not protective in both the cell culture and *ex vivo* pemphigus skin models, when a PV-IgG from a different patient (PV4-IgG), containing high levels of anti-Dsg1 autoantibodies was used.

## Results

### Inhibition of ADAM10 Strengthened Cell-Adhesion in Primary Human Keratinocytes

**Figure 1:**
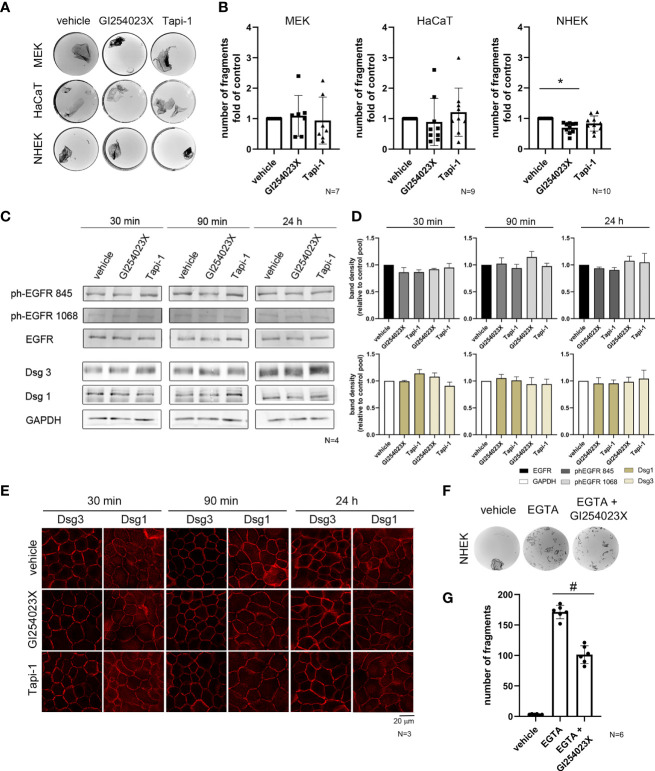
ADAM10 inhibition improved cell adhesion in primary keratinocytes and induces hyperadhesion. **(A)** Dispase-based keratinocyte dissociation assays were performed by applying shear stress on epidermal monolayers. Images show the fragmentation of cell monolayers for three different epithelial keratinocyte cell types with or without inhibition of ADAM10 (GI254023X) and ADAM17 (Tapi-1) for 24 h. **(B)** Quantification of the dissociation assay for MEK cells and HaCaT cells showed no significant difference between control conditions (vehicle), and the incubation with ADAM10-inhibitor (*p* > 0.99), whereas in NHEK cells, inhibition of ADAM10 by GI254023X reduced cell sheet fragmentation significantly (*p = 0.001). In contrast, there was no effect of inhibition of ADAM17 in all cell types (*p* > 0.99) (n≥7). Western blot analysis **(C)** and its quantification **(D)** showed no significant difference in total protein amount of Dsg1 and Dsg3. GAPDH was used as a loading control. There was no significant activation of EGFR at Tyr845 and Tyr1068 in comparison to the total amount of EGFR (n = 4). **(E)** Immunostaining for the desmosomal proteins Dsg1 and Dsg3 showed no remarkable difference, due to the inhibition of ADAM10 (GI254023X) and ADAM17 (Tapi-1) compared to the respective control condition (vehicle). **(F)** Hyperadhesion dissociation assays in NHEK were performed. Inhibition of ADAM10 enhanced cell adhesion after incubation with the Ca^2+^chelator EGTA. **(G)** Quantification of hyperadhesion dissociation assay showed a significant reduction of cell sheet fragments after inhibition of ADAM10 (^#^ *p* < 0.0001; n = 6).

Since molecules related to metalloproteinases such as ADAM5, ADAM10, ADAM15 and ADAM17, can affect cell adhesion (8) and ADAM10 and ADAM17 are involved in the turnover of Dsg2 (17, 24, 31), we investigated the effects of ADAM10 and ADAM17 on intercellular adhesion of keratinocytes using specific inhibitors. First, we performed dispase-based dissociation assays to analyze the intercellular adhesion of immortalized murine (MEK) and human (HaCaT) as well as primary human (NHEK) epidermal keratinocytes (**Figures 1A, B**). Inhibition of ADAM17 by Tapi-1 for 24 h showed no significant effect in all cell types. Treatment of MEK and HaCaT cells with ADAM10 inhibitor GI254023X showed no difference in cell sheet fragmentation, either. In contrast, GI254023X significantly strengthened cell adhesion in primary human NHEK cells under basal conditions (**Figure 1B**).

ADAMs were shown to regulate shedding of Dsg2 (31, 32) which is almost absent in healthy adult human skin (33, 34). Thus, desmosomal adhesion in the skin is maintained by desmosomal cadherins other than Dsg2. Therefore, we speculated that ADAMs might also regulate adhesion of the pemphigus antigens Dsg1 and Dsg3. Further, besides the release of cell adhesion, ADAM10 is a main sheddase of EGF and ADAMs have a critical role in releasing EGFR ligands. Since inhibition of ADAM10 under basal conditions in NHEK cells showed a stabilizing effect, we tested whether inhibition of ADAMs changed protein levels of Dsg1 and Dsg3 or the activation status of EGFR on Tyrosine (Tyr) 1068 and the Src-dependent phosphorylation side Tyr845 would change in response to inhibition of ADAMs. Thus, we performed Western blotting experiments following incubation with both ADAM inhibitors, Tapi-1 and GI254023X for 30 min, 90 min and 24 h (**Figure 1C**), which did affect neither protein levels of Dsg1 and Dsg3 nor activation of EGFR (**Figure 1D**). Immunostaining revealed no changes in Dsg3 and Dsg1 localization after incubation with both ADAM inhibitors at the respective time points, neither at cell borders nor in the cytoplasm (**Figure 1E**).

### ADAM10 Inhibition Shifted Keratinocyte Adhesion Towards a Hyperadhesive State

The skin is exposed to high mechanical stress, and its strong intercellular adhesion is mediated by desmosomes (3, 35–37). In the mature epidermis most of the desmosomes were characterized as hyperadhesive (38–41). Hyperadhesion is thought to be a strong adhesive state in which desmosomes become independent of Ca^2+^, and is important for strong cell adhesion and resistance to high mechanical stress (41). Keratinocytes acquire a hyperadhesive state at a distinct time point of maturation (38). Because ADAM10 inhibition enhanced cell cohesion in NHEK cells cultured under basal conditions, we performed a dispase-based dissociation assay to test hyperadhesive conditions in human primary epithelial keratinocytes (**Figure 1F**). After incubation with or without the ADAM10-inhibitor GI254023X for 24 h, cells were detached by dispase, subjected to the Ca^2+^chelator EGTA for 90 min and subsequently exposed to defined mechanical stress. In this dissociation assay, GI254023X significantly reduced the loss of cell cohesion (**Figure 1G**), indicating that ADAM10 regulates cell adhesion and contributes to hyperadhesion.

### ADAM10 Inhibition Ameliorated Loss of Cell Adhesion Induced by the Dsg3-Specific Autoantibody AK23 and by PV-IgG Containing Less Anti-Dsg1 Autoantibodies *In Vitro*

**Figure 2:**
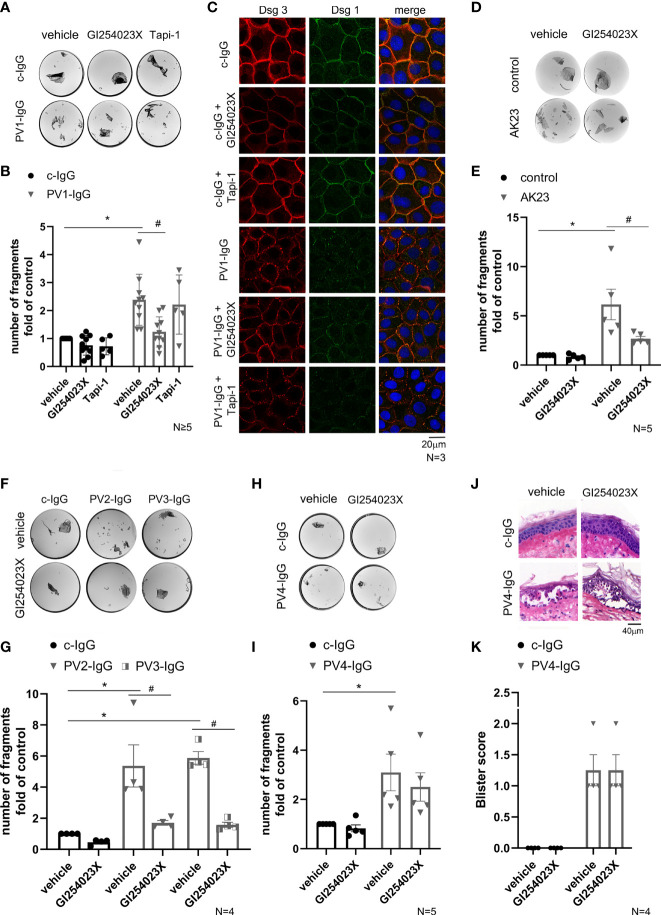
ADAM10 inhibition ameliorated loss of intercellular adhesion in response to PV-IgGs with low Dsg1-levels and AK23. **(A)** Representative images of dissociation assays showed loss of cell adhesion in NHEK caused by PV-IgG compared to control autoantibodies from healthy volunteers (c-IgG). PV1-IgG in combination with inhibition of ADAM10 showed reduced cell sheet fragmentation. In contrast to ADAM10 inhibition, no protective effect was observed when PV1-IgG was co-incubated with the ADAM17 inhibitor. **(B)** Quantification of the assay showed a significant increase of cell sheet fragmentation after incubation with PV1-IgG (*p˂0.0001) compared to the control condition (vehicle of c-IgG) and a reduction of cell fragments after co-incubation with GI254023X (#p=0.0016; n≥5) compared to PV1-IgG alone. **(C)** Immunostaining under the same conditions showed a fragmented immunostaining of Dsg3 and Dsg1 after incubation with PV1-IgG which was ameliorated by ADAM10 inhibitor. The effect of ADAM10 inhibition was not detectable in cells incubated with Tapi-1. DAPI-staining proved the number and vitality of cells (n=3, scale bar: 20 µm). **(D)** Involvement of Dsg3 in this process was supported by analysis of the cell cohesion with incubation of AK23 in a dispase-based assay. **(E)** Quantification revealed the protective effect of GI254023X (#p=0.0387) vs. PV-IgG alone. Number of fragments were normalized to the control condition: vehicle of control (n=5). **(F, G)** Dispase assays after incubation with the mucosal PV2-IgG and PV3-IgG for 24 h leads to cell-cell fragmentation (PV2-IgG-vehicle: *p=0.0008 vs. c-IgG-vehicle and PV3-IgG-vehicle: *p=0.0002 vs. c-IgG-vehicle). Co-incubation with GI254023X revealed the protective effect of ADAM10 inhibition, when anti-Dsg1 autoantibodies are almost not present (PV2-IgG-vehicle vs. PV2-IgG-GI254023X: #p=0.0049 and PV3-IgG-vehicle vs. PV3-IgG-GI254023X: #p=0.0009). Number of fragments were normalized to c-IgG-vehicle. (n=4). **(H)** Representative images of a dissociation assay with PV4-IgG in combination with or without ADAM10 inhibitor. **(I)** After incubation with PV4-IgG for 24 h, a significant increase of cell fragments was detectable (*p=0.0401) which was not reduced by the ADAM10 inhibitor. Number of fragments were normalized to the control condition: vehicle of c-IgG (n=5). **(J)** Human skin samples were injected with PV4-IgG with or without GI254023X. HE staining of the skin samples showed no effect of GI254023X on PV4-IgG-induced acantholysis. **(K)** Blister score reveals no difference in absence or presence of ADAM10 (n=4).

Furthermore, we investigated whether inhibition of ADAMs also play a role in human keratinocyte models for pemphigus, a blistering disease in which autoantibodies mainly against Dsg1 and Dsg3 lead to a loss of cell adhesion. Therefore, we examined cell cohesion in NHEK after incubation with PV-IgG from a PV patient (designated PV1-IgG) for 24 h in comparison to control IgG (c-IgG) from healthy volunteers (**Figure 2A**). Co-treatment with GI254023X significantly diminished cell sheet fragmentation whereas ADAM17 inhibition did not (**Figures 2A, B**). To investigate the effects of ADAM inhibition on PV1-IgG treatment with respect to desmosome organization, we performed immunostaining against Dsg3 and Dsg1 in NHEK cells (**Figure 2C**). Under control conditions with or without ADAM10- and ADAM17-inhibition, Dsg3 and Dsg1 revealed a linear, continuous staining along cell borders. PV1-IgG induced fragmentation and reduced membrane staining of Dsg3 and Dsg1, which was referred to as Dsg depletion (42–45). In line with the dissociation assay, inhibition of ADAM10, but not ADAM17, ameliorated PV1-IgG-induced Dsg reorganization at cell-cell borders. To test whether the protective effect of ADAM10 inhibition depends on the amount of autoantibodies targeting Dsg3, we performed dissociation assays with AK23, a pathogenic monoclonal Dsg3 autoantibody derived from a pemphigus mouse model (46). Indeed, co-treatment with GI254032X for 24 h significantly diminished AK23-induced loss of adhesion (**Figures 2D, E**). Next, we exposed keratinocytes to two mucosal PV-IgG fractions (designated PV2-IgG and PV3-IgG) containing none anti-Dsg1 autoantibodies (cut off value: 20 U/ml) with or without GI254023X. Interestingly, cell monolayer fragmentation after a dispase-based assay was significantly ameliorated by co-incubation with the ADAM10 inhibitor in comparison to the respective PV-IgG alone (**Figures 2F, G**).

### ADAM10 Inhibition Was Not Effective to Stabilize Cell Adhesion After Treatment With a PV-IgG With High Level of Dsg1 and Dsg3 Antibodies *In Vitro* and *Ex Vivo*

It is known that each pemphigus patient has a distinct autoantibody profile, which leads to different intracellular signaling pattern (15, 30). Given that PV1-IgG contained more than 200 U/ml of anti-Dsg3-antibodies but only 59 U/ml of anti-Dsg1 autoantibodies, and PV2-IgG and PV3-IgG were from patient with mucosal PV, we repeated the dissociation assay with a PV-IgG from another mucocutaneous PV patient (designated PV4-IgG). PV4-IgG contained a higher level of antibodies against Dsg1 (167.9 U/ml) and a comparable level of autoantibodies against Dsg3 (185.8 U/ml). However, there was no significant reduction of fragments by co-incubation with ADAM10 inhibitor in dissociation assays compared to PV4-IgG alone (**Figures 2H, I**).

To substantiate that ADAM10 inhibition is not effective to preserve cell adhesion against PV-IgG including a high level of autoantibodies against Dsg1 in intact human skin, we used the human *ex vivo* skin model (**Figures 2J, K**). As reported previously, we injected PV4-IgG to induce pemphigus typical acantholysis in human skin (15, 47, 48). Samples were incubated for 24 h and subsequently subjected to mechanical stress. HE-stained sections revealed blister formation after treatment for 24 h with PV4-IgG alone. ADAM10 inhibitor did not prevent PV4-IgG-induced intraepidermal blistering in human skin *ex vivo* (**Figures 2J, K**). Taken together, the involvement of ADAM10 in PV pathogenesis seems to be dependent on the patient and autoantibody profile.

## Discussion and Perspectives

We addressed the role of the sheddases ADAM10 and ADAM17 in both keratinocyte adhesion and loss of cell adhesion in pemphigus in human keratinocytes. The results indicated that an inhibition of ADAM10 increases cell adhesion in primary human keratinocytes. ADAM10 was shown to cleave Dsg2 and thereby regulate Dsg2 turnover (18, 31, 32). Moreover, ADAM10 inhibition was shown to be protective in a murine pemphigus model when antibodies against Dsg1 and Dsg3, but not Dsc3, were present in the patient sera (30).

Given that Dsg2 is almost absent in healthy human skin (33, 34), in the present study we analyzed the effect of ADAMs on the two main targets of autoantibodies in PV, i.e. Dsg1 and Dsg3. However, inhibition of ADAM10 and ADAM17 did not change either the Dsg1 and Dsg3 protein levels or the activation state of EGFR under basal conditions.

Interestingly, ADAM10 inhibition shifted keratinocyte adhesion towards a hyperadhesive state, which is suggested to be required for keratinocytes and cardiomyocytes to acquire a very strong adhesive state in order to resist a high mechanical stress and probably reduce susceptibility for diseases (38, 49, 50). This is in line with the finding that ADAM10 is more active at high Ca^2+^ (23, 28). These results in this study are novel and open new perspectives to study regulation of hyperadhesion.

Desmosomes are known to be dynamic structures in a constant process of assembly and disassembly. Trapping of desmosomal components inside of desmosomes by enhancing their cytoskeletal anchorage, a process controlled *via* desmoplakin phosphorylation, was proposed to lead to hyperadhesion (51, 52). This mechanism was also shown to render keratinocytes protected against PV-IgG-induced loss of adhesion (53, 54). However, the mechanisms controlling hyperadhesion in keratinocytes are not fully understood, although hyperadhesion may be related to the observation that ADAM10 inhibition was not sufficient to ameliorate loss of adhesion for PV-IgGs in all PV patients. We found that ADAM10 inhibition is protective against the pathogenic effects of pemphigus autoantibodies which primarily target Dsg3 but not Dsg1. This is in line with the result that the binding strength and clustering of Dsg3 molecules increased strongly during desmosomal hyperadhesion, whereas Dsg1 remained unaffected (55). Other desmosomal plaque proteins such as plakophilin 1 (PKP1) and plakophilin 3 (PKP3), may also be important for control of Dsg3 clustering.

Interestingly, ADAM inhibition ameliorated loss of cell adhesion induced by PV-IgGs with high levels of Dsg3 antibodies but lesser levels of Dsg1 antibodies and the murine, pathogenic Dsg3 antibody AK23. In contrast ADAM10 inhibition was not protective when PV-IgG with higher Dsg1 antibody levels were used. It is known that antibodies against Dsg1 are required to induce blistering in human skin and loss of Dsg1 in a mouse model showed a lethal skin blistering phenotype (56, 57) and therefore Dsg1 plays an important role in mucocutaneous pemphigus.

Further, it was shown that excessive shedding of the hemidesmosomal transmembrane protein and major autoantigen in bullous pemphigoid, Collagen XVII (also referred to as 180-kd bullous pemphigoid antigen (BP180)), by ADAMs is linked to reduced adhesion and blister formation in this disease (29). Furthermore, a case report showed a neonatal PV patient with no anti-Dsg1-autoantibodies but a high levels of autoantibodies targeting BP180 (58).

Taken together, these results indicate that ADAM10 primarily controls Dsg3-mediated cell adhesion. This would be in line with the concept that ADAM10 together with Src regulates signaling downstream of Dsg3 which may also include signaling by EGFR (30, 59).

## Concluding Remarks

We demonstrated for the first time that ADAMs are involved in hyperadhesion, which may contribute to strengthen Dsg3-mediated cell adhesion. However, the protective effect of ADAM10 inhibition in pemphigus appears to be dependent on the autoantibody-profile of different pemphigus patients, which is in line with a previous study (30). In addition, we showed a protective effect of ADAM10 inhibition in a Dsg3-specific manner. Here, we demonstrated for the first time the role of ADAM10 inhibition in primary human keratinocytes and human *ex vivo* skin samples. In contrast to the study of Ivars at al. (30), we conclude that ADAM10 inhibition is not protective against PV-IgGs with a high level of Dsg1 autoantibodies and therefore the inhibition of ADAMs may be not sufficient for the treatment of all PV patients but may be a promising approach for treatment of mucosal PV.

## Data Availability Statement

The raw data supporting the conclusions of this article will be made available by the authors, without undue reservation.

## Ethics Statement

PV sera from patients were reviewed and approved by the ethics approval Az. 20/14, University of Marburg, Germany; Decision No. 48825-5/2019/EÜIG of the National Health Centre, Hungary and by the ethics approval number 127, approved by Kurume University Ethics Committee, Japan. NHEKs were generated at the Universitäts-Hautklinik Tübingen, under abidance to the treaty of Helsinki (ethics approval: 547/2011BO2). The patients/participants provided their written informed consent to participate in this study.

## Author Contributions

DK, MA, AMS and DE performed the experiments. JW and DK contributed to the conception and design of the study. JW and DK interpreted the data and wrote the manuscript. RAE and ASY provided the NHEK cells. MS, DD, MH and TH examined PV-patient, evaluated and provided the sera from pemphigus patients. All authors contribute to manuscript, read and approved the final version.

## Funding

This work was supported by DFG FOR 2497 TP5 to JW and WiFoMed (Verein zur Förderung von Wissenschaft und Forschung an der Medizinischen Fakultät der LMU München e.V.) to DK.

## Conflict of Interest

The authors declare that the research was conducted in the absence of any commercial or financial relationships that could be construed as a potential conflict of interest.

## Publisher’s Note

All claims expressed in this article are solely those of the authors and do not necessarily represent those of their affiliated organizations, or those of the publisher, the editors and the reviewers. Any product that may be evaluated in this article, or claim that may be made by its manufacturer, is not guaranteed or endorsed by the publisher.

## Abbreviations

ADAM10, a disintegrin and metalloproteinase domain-containing protein 10; ADAM17, a disintegrin and metalloproteinase 17; AK23, monoclonal Dsg3 autoantibody; BP180, 180-kd bullous pemphigoid antigen; Dsg1, desmoglein 1; Dsg3, desmoglein 3; EGFR, epidermal growth factor receptor; IgG, immunoglobulin; PF, pemphigus foliaceus; PV, pemphigus vulgaris; PV-IgG, autoantibodies from pemphigus vulgaris patients; c-IgG, autoantibody-control from healthy people.
