# Supplementary material for: Role of ADAM10 and ADAM17 in the Regulation of Keratinocyte Adhesion in Pemphigus Vulgaris
Source: Front Immunol. 2022 Jun 30;13:884248. doi: 10.3389/fimmu.2022.884248 (PMC9279611; doi:10.3389/fimmu.2022.884248)
Supplement: Supplementary file 1 [file DataSheet_1.docx]

**Supplementary material**

# Material and Methods

**Cell Culture**

The immortalized human keratinocyte cell line HaCaT was cultured in Dulbecco´s Modified Eagle Medium (DMEM) (Life Technologies, Carlsbad, CA) supplemented with 10% FCS (Biochrom, Berlin, Germany), 50 U/ml penicillin and 50 µg/ml streptomycin (both AppliChem, Darmstadt, Germany). The normal human epidermal keratinocytes cells (NHEK) were generated at the Universitäts-Hautklinik Tübingen, under abidance to the treaty of Helsinki (ethics approval: 547/2011BO2). The cells were cultured in Epithelial Proliferation Medium (CnT-07) with the supplemental pack provided by the company (CellnTEC, Bern, Switzerland). After reaching confluence, cells were switched to 1.8 mM Ca^2+^ and used for experiments after 24 h. Both cell lines were cultivated in a humidified atmosphere of 5% CO_2_ at 37°C.

**Preparation and cell culture of mouse keratinocytes**

Murine keratinocytes from the epidermis of newborn mice (MEK) were isolated and immortalized according to the literature for preparation of mouse keratinocytes^1-3^. In brief, the skin was taken and incubated for 16 h in 2.4 U/ml dispase II in PBS supplemented with Gentamicin/AmphotericinB (CELLnTEC, Bern, Switzerland) at 4°C. After separating the dermis and epidermis, the epidermis was incubated for 20 min with accutase (CELLnTEC, Bern, Switzerland) at room temperature, in order to dissociate the cells. Mouse keratinocytes were resuspended and then grown in complete FAD medium (0.05 mM CaCl_2_) on collagen I-coated culture dishes (rat tail; BD Bioscience, New Jersey, USA). The cells were cultivated in a humidified atmosphere containing 5% CO_2_ at 35°C. After reaching confluence, cells were switched to 1.2 mM Ca^2+^ and used for experiments after 48 h.

**Test reagents, antibodies and purification of PV-IgG fractions**

The ADAM10-inhibitor GI254023X (Sigma-Aldrich, St. Louis, MO, U.S.A) was used at 20 µM and the ADAM17-inhibitor Tapi-1 (Cayman Chemicals, Ann Arbor, U.S.A.) was used at 10 µM for the respective time periods. The following commercial primary antibodies were used for Western blot and immunofluorescence analyses: anti-EGF-receptor (EGFR) antibody (Cell Signaling, Danvers/Massachusetts, U.S.A.), anti-phospho-EGFR Tyr845 antibody (Cell Signaling, Danvers/Massachusetts, U.S.A.), anti-phospho-EGFR Tyr1068 antibody (Cell Signaling, Danvers/Massachusetts, U.S.A.), anti-Dsg3 antibody (Elabscience/Biozol, Eching, Germany), anti-Dsg1 antibody (Abclonal/Biozol, Eching, Germany), glycerinaldehyd-3-phosphate-dehydrogenase (GAPDH) (Santa Cruz, Heidelberg, Germany). The corresponding secondary antibodies for immunofluorescence analysis were purchased from Dianova (Hamburg). 4’.6-diamidino-2-phenylindole (DAPI) (Roche, Basel, Switzerland) was used to determine the number of nuclei and to assess the cell viability. AK23, a monoclonal pathogenic autoantibody, derived from a pemphigus mouse model, was purchased from Biozol (Eching, Germany) and used at 75 µg/ml. PV-IgGs and IgG fractions pooled from three healthy donors (c-IgG) were purified as described previously^4^. For purification, 2 ml of patient sera were solved in 1 ml PBS, this was used in a 1:50 dilution in the dispase based keratinocyte dissociation assay or purely injected for the ex vivo skin model. Patients and donors gave written consent for research use. The autoantibody profiles were determined using enzyme-linked immunosorbent assays (ELISAs) (MBL, Japan). ELISA scores of antibodies against Dsg1 and Dsg3 were determinded before purification (cut-off value: 20 U/ml). The following scores for the desmosomal autoantibodies from four PV patients were detected: PV1-IgG: Dsg1: 59 (+) U/ml; Dsg3: > 200 (+) U/ml; PV2-IgG: Dsg1: 14.4 U/ml; Dsg3 162.9 U/ml; PV3-IgG: Dsg1: 1.4 U/ml; Dsg3: 154.0 U/ml and PV4-IgG: Dsg1: 167.9 U/ml; Dsg3 185.8 U/ml. A positive vote of the Ethics Committee from the Medical Faculty of the University of Marburg, Germany (Az 20/14), Budapest, Hungary (48825-5/2019/EÜIG) and Kurume, Japan (No. 127) was given.

**Western blotting**

Cells were washed with PBS, lysed with SDS-lysis buffer (25 mmol/l HEPES, 25 mmol/l NaF and 1% SDS, pH 7.4) and sonicated on ice. Protein amounts were determined using the Pierce^TM^ BCA Protein Assay Kit (Thermo Fisher, USA). Cell lysates were mixed with laemmli buffer containing 50 mM dithiothreitol. Electrophoresis and western blotting were performed according to standard procedures. Membranes were incubated at 4°C overnight with respective primary autoantibody in tris-buffered-saline containing 0.05% tween (TBS-T), and supplemented with 5% bovine serum albumin (BSA).

**Histology and immunostaining**

Human tissue samples were embedded in Tissue Tec (Leica Biosystems, Nussloch, Germany) and thereafter serial-sectioned at 7 µm thickness using a cryostat microtome (Cryosstar NX70, Thermo Fisher). Hematoxylin and esoin (H.E.) staining was performed according to standard protocols and mounted in DEPX (Sigma-Aldrich, St. Louis, MO, U.S.A). Images were captured using a Leica DMi8 microscope with a HC PL APO 40x/0.85 dry objective. For immunofluorescence analysis, cells were seeded onto coverslips and grown to confluence. After respective treatment, cell monolayers were washed with PBS and thereafter fixed and permeabilzed with ethanol for 30 min on ice, following the treatment with acetone for 3 min on ice. Next, samples were rinsed three times with PBS and thereafter blocked with 3% bovine serum albumin and 1% normal goat serum for 60 min. The primary antibodies were incubated overnight at 4°C. After washing with PBS, respective secondary antibodies were applied for 60 min at room temperature. Subsequently, coverslips were washed and mounted with 1.5% n-propyl gallate in glycerol. Specificity of staining were tested by secondary antibody controls. Images were taken with a Leica SP5 confocal microscope using a 63x/1.40 PL APO oil objective (Leica, Mannheim, Germany).

**Dispase-based dissociation assay**

After incubation with test reagents, confluent cell monolayers of cultured cells were washed with Hank´s buffered saline solution (HBSS) (Sigma-Aldrich, St. Louis, MO, U.S.A) and subjected to 2.4 U/ml dispase II (Sigma-Aldrich, St. Louis, MO, U.S.A) in HBSS for 20 min at 37°C and 5% CO_2_. After dispae-based detachment of the monolayer, the reaction was stopped by replacing the dispase II solution with HBSS. Defined shear stress was applied, by pipetting with a 1 mL electrical pipette. Resulting fragments were stained with the thiazolyl blue tetrazolium bromide dye MTT (Sigma-Aldrich, St. Louis, MO, U.S.A) and counted using a binocular microscope (Leica, Mannheim, Germany). All independent experiments were performed in duplicates. The quantification of the dissociation assay fragments was shown as fold of control, for that, the respective control condition was normalized to 1.

**Hyperadhesion keratinocyte dissociation assay**

NHEK cells reached 48 h hours after confluence hyperadhesive state, thereafter the cells were incubated with the respective mediators for 24 h hours, thereafter cell monolayers were detached from the well bottom by a mixture of Dispase II as described in the previous section. When monolayers were ﬂoating, enzymes were removed and substituted by Epithelial Proliferation Medium (CnT-07). EGTA at a concentration of 2.5 mM was added for 90 min at a humidified atmosphere of 5% CO_2_ at 37°C. Afterwards defined shear stress was applied with an electrical pipette and fragments were counted using a binocular microscope (Leica, Mannheim, Germany). All independent experiments were performed in duplicates.

**Ex vivo human skin model**

Biopsies of healthy human skin were acquired from cadavers from the human body donor program from the institute of Anatomy and Cell Biology, Ludwig-Maximilians-Universität München, Germany. Written informed consent was given from body donors for the use of research samples. Biopsies were taken only if death occurred less than 24 h before arrival at the institute. From each body donor, a skin piece of approximately 5 x 5 cm size was removed from the shoulder, gently stripped off fat including excessive connective tissue. The skin was cut into 1 cm^2^ pieces and injected intradermally with 50 µl of the respective IgG-fraction (c-IgG and PV-IgG) with or without Adam 10 inhibitor GI254023X in a concentration of 20 µM, using a 30 G syringe. Next, samples were incubated floating on DMEM at 37°C and 5% CO_2_ for 24 h. After incubation sheer stress was applied using a rubber head with equal frequency and magnitude. Treated samples were embedded in tissue tec, cut and processed for hematoxylin and eosin (HE) staining. Blister score of the human samples was measured as described below.

**Scoring of blister size**

Each section was evaluated and sorted into the following score system as published previously^5^: Absence of intraepidermal separation, score 0; cleft size covering 1%-25% of total section length, score 1; cleft size between 26%-50% of section length, score 2; cleft size between 51%-75% section length, score 3; and cleft size between 76%-100%, score 4.

**Statistical analysis**

Images were processed using Photoshop CC (Adobe Systems, San Jose, CA). Statistical analysis was performed using one way ANOVA or two way ANOVA respectively followed by Bonferroni correction using Graphpad Prism (Graphpad Software, LaJolla, CA). Significance was presumed with p ≤ 0.05. Data are shown as mean ± SEM. Each n represents one independent experiment.

# Reference

1 Lichti, U., Anders, J. & Yuspa, S. H. Isolation and short-term culture of primary keratinocytes, hair follicle populations and dermal cells from newborn mice and keratinocytes from adult mice for in vitro analysis and for grafting to immunodeficient mice. *Nat Protoc* **3**, 799-810, doi:10.1038/nprot.2008.50 (2008).

2 Kroger, C. *et al.* Keratins control intercellular adhesion involving PKC-alpha-mediated desmoplakin phosphorylation. *J Cell Biol* **201**, 681-692, doi:10.1083/jcb.201208162 (2013).

3 Kumar, V. *et al.* A keratin scaffold regulates epidermal barrier formation, mitochondrial lipid composition, and activity. *J Cell Biol* **211**, 1057-1075, doi:10.1083/jcb.201404147 (2015).

4 Waschke, J., Bruggeman, P., Baumgartner, W., Zillikens, D. & Drenckhahn, D. Pemphigus foliaceus IgG causes dissociation of desmoglein 1-containing junctions without blocking desmoglein 1 transinteraction. *J Clin Invest* **115**, 3157-3165, doi:10.1172/JCI23475 (2005).

5 Spindler, V. *et al.* Peptide-mediated desmoglein 3 crosslinking prevents pemphigus vulgaris autoantibody-induced skin blistering. *J Clin Invest* **123**, 800-811, doi:10.1172/JCI60139 (2013).
